# Discovering joint associations between disease and gene pairs with a novel similarity test

**DOI:** 10.1186/1471-2156-11-86

**Published:** 2010-10-04

**Authors:** Wan-Yu Lin, Wen-Chung Lee

**Affiliations:** 1 Institute of Epidemiology and Preventive Medicine, College of Public Health, National Taiwan University, No. 17, Xuzhou Rd., Taipei 100, Taiwan; 2 Department of Biostatistics, University of Alabama at Birmingham, 1665 University Boulevard, Birmingham, Alabama 35294, USA; 3 Research Center for Genes, Environment and Human Health, National Taiwan University, No. 17, Xuzhou Rd., Taipei 100, Taiwan

## Abstract

**Background:**

Genes in a functional pathway can have complex interactions. A gene might activate or suppress another gene, so it is of interest to test joint associations of gene pairs. To simultaneously detect the joint association between disease and two genes (or two chromosomal regions), we propose a new test with the use of genomic similarities. Our test is designed to detect epistasis in the absence of main effects, main effects in the absence of epistasis, or the presence of both main effects and epistasis.

**Results:**

The simulation results show that our similarity test with the matching measure is more powerful than the Pearson's *χ*^2 ^test when the disease mutants were introduced at common haplotypes, but is less powerful when the disease mutants were introduced at rare haplotypes. Our similarity tests with the counting measures are more sensitive to marker informativity and linkage disequilibrium patterns, and thus are often inferior to the similarity test with the matching measure and the Pearson's *χ*^2 ^test.

**Conclusions:**

In detecting joint associations between disease and gene pairs, our similarity test is a complementary method to the Pearson's *χ*^2 ^test.

## Background

Genes in a functional pathway can have complex interactions. A gene might activate or suppress another gene, so it is of interest to test joint associations of gene pairs. Differing from *epistasis *(generally defined as the *interaction *between different genes [1]), *joint associations *herein include both main effects and interactions. Haplotypes from two receptors can trigger significant interactions affecting disease status [2]. Moreover, detecting associations with the use of haplotypes constructed by several adjacent and highly correlated single-nucleotide polymorphisms (SNPs) is an economical strategy. These all enlighten us regarding ways to develop methods for discovering gene pairs in association with disease by using haplotypes.

There is a growing interest in detecting gene-gene interactions [1,3,4], and some methods have been proposed to detect interactions. A well-known approach to detecting SNP-SNP interactions, the multifactor dimensionality reduction (MDR) method [5-8], however, has not been developed for testing haplotype-haplotype interactions. Another commonly used method is the classification and regression trees (CART) [9-12]. This concept has been extended to analyze haplotype data, known as the *HapForest *approach [13].

In this paper, we do not focus only on *interactions *because the definition of independence between two genes is arbitrary, often varying according to the field under discussion, such as biology, statistics or epidemiology [1]. Instead, we focus on detecting *joint associations*. To simultaneously detect joint association between disease and two genes (or two chromosomal regions), we propose a new test with the use of genomic similarities. Similarity-based methods are less vulnerable to the penalty of testing many markers or haplotypes, and can be more powerful than conventional association methods in some situations [14]. Our proposed test is designed to detect epistasis in the absence of main effects, main effects in the absence of epistasis, or the presence of both main effects and epistasis. We further compare our method with the *HapForest *approach [13], the Pearson's *χ*^2 ^test, and the tests for SNP × SNP epistasis via simulation studies.

## Methods

### Similarity Measures

Let $S_{ij}^{G_{1}}$ and $S_{ij}^{G_{2}}$ be the marginal similarities of the *i*^th ^and *j*^th ^subjects at genes *G*_1 _and *G*_2_, respectively. They can be obtained based on unphased multi-marker genotypes or statistically inferred haplotypes, and they can be scaled from 0 to 1. Here we list some commonly used similarity measures, which can be traced back to [15,16].

#### A. Diplotype perspective

A.1. Similarity measure based on identity-by-state (IBS) allele sharing (referred to as 'IBS'):

$$S_{ij}^{G_{k}}=\frac{\sum_{l=1}^{L}s(g_{i}^{k,l},g_{j}^{k,l})}{2L},$$

where *L *is the number of loci considered in *G_k_*; $g_{i}^{k,l}$ and $g_{j}^{k,l}$ are respectively the genotypes of the *i*^th ^and *j*^th ^subjects at the *l*th locus in *G_k_*; $s(g_{i}^{k,l},g_{j}^{k,l})$ is the number of alleles shared in common for the *i*^th ^and *j*^th ^subjects at the *l*th locus in *G_k_*, which has possible values of 0, 1, and 2.

A.2. Similarity measure based on IBS inversely weighted by genotype frequencies (referred to as 'W-IBS'):

$$S_{ij}^{G_{k}}=\frac{\sum_{l=1}^{L}w_{ij}^{k,l}s(g_{i}^{k,l},g_{j}^{k,l})}{\sum_{l=1}^{L}w_{ij}^{k,l}},$$

where $w_{ij}^{k,l}={\left[f(g_{i}^{k,l})\cdot f(g_{j}^{k,l})\right]}^{-1}$, and $f(g_{i}^{k,l})$ is the frequency of genotype $g_{i}^{k,l}$. The implication of this weight is that subjects sharing rare alleles may have more similar genomes than do subjects sharing common alleles.

#### Joint Similarity Regarding Two Genes

A similarity measure accounting for the joint association of genes *G*_1 _and *G*_2 _for the *i*^th ^and *j*^th ^subjects is

(1) $$S_{ij}^{G_{1},G_{2}}=S_{ij}^{G_{1}}\times S_{ij}^{G_{2}},$$

where $S_{ij}^{G_{1},G_{2}}$ ranges from 0 to 1, too. The joint similarity ($S_{ij}^{G_{1},G_{2}}$) will be high if both of the two marginal similarities ($S_{ij}^{G_{1}}$ and $S_{ij}^{G_{2}}$) are high. That is, with respect to the two genes, the *i*^th ^and *j*^th ^subjects will be regarded as 'similar' if they are similar in both genes.

#### B. Haplotype perspective

B.1. Similarity based on the counting measure for haplotypes (referred to as 'COUNT'):

Let *h_i _*and *h_j _*be the *i*^th ^and *j*^th ^categories of haplotypes in a gene, $h_{i}^{l}$ and $h_{j}^{l}$ are the alleles at the *l*th locus on *h_i _*and *h_j_*, respectively. The similarity based on the counting measure for haplotypes is

$$S_{h_{i},h_{j}}=\frac{\sum_{l=1}^{L}s(h_{i}^{l},h_{j}^{l})}{L},$$

where $s(h_{i}^{l},h_{j}^{l})$ is 1 if the alleles at the *l*th locus match for the *i*^th ^and *j*^th ^haplotypes.

B.2. Similarity based on the matching measure for haplotypes (referred to as 'MATCH'):

Let *h_i _*and *h_j _*be the *i*^th ^and *j*^th ^categories of haplotypes in a gene, then the similarity based on the matching measure for haplotypes is

$$S_{h_{i},h_{j}}=s(h_{i},h_{j}),$$

where *s*(*h_i_*, *h_j_*) is 1 only when *all *alleles match for the *i*^th ^and *j*^th ^haplotypes, otherwise *s*(*h_i_*, *h_j_*) is 0.

#### Joint Similarity Regarding Two Genes

Let $h_{iu}^{k}=(h_{iu1}^{k}/h_{iu2}^{k})$ be the *u*^th ^possible diplotype (i.e., the pair of haplotypes a subject possesses) in *G_k _*of the *i*^th ^subject, where $u=1,\cdots ,n_{h_{i}^{k}}$, and where $n_{h_{i}^{k}}$ is the number of possible diplotypes in *G_k _*for the *i*^th ^subject. $P(h_{iu}^{k}|g_{i}^{k})$ is the posterior probability that the *i*^th ^subject has the *u*^th ^possible diplotype in *G_k_*, given the unphased genotypes ($g_{i}^{k}$). $P(h_{iu}^{k}|g_{i}^{k})$ can be inferred by the expectation-maximization (EM) algorithm [17]. Then a similarity measure accounting for the joint association of genes *G*_1 _and *G*_2 _for the *i*^th ^and *j*^th ^subjects is

(2) $$S_{ij}^{G_{1},G_{2}}=\sum_{u}\sum_{v}\sum_{y}\sum_{z}\sum_{m=1}^{2}\sum_{n=1}^{2}\sum_{p=1}^{2}\sum_{q=1}^{2}P(h_{iu}^{1}|g_{i}^{1})\cdot P(h_{jv}^{1}|g_{j}^{1})\cdot P(h_{iy}^{2}|g_{i}^{2})\cdot P(h_{jz}^{2}|g_{j}^{2})\cdot S_{h_{ium}^{1},h_{jvn}^{1}}\cdot S_{h_{iyp}^{2},h_{jzq}^{2}},$$

where $S_{h_{ium}^{1},h_{jvn}^{1}}$ and $S_{h_{iyp}^{2},h_{jzq}^{2}}$ can be obtained based on the counting measure or the matching measure. $S_{ij}^{G_{1},G_{2}}$ ranges from 0 to 1, too.

### Similarity Test

Let the dissimilarity accounting for the joint association of genes *G*_1 _and *G*_2 _for the *i*^th ^and *j*^th ^subjects be $D_{ij}^{G_{1},G_{2}}=1-S_{ij}^{G_{1},G_{2}}$. The test statistic to detect the joint association of genes *G*_1 _and *G*_2 _is

(3) $$\begin{array}{c} T=\frac{\frac{1}{n_{CS}\times n_{CN}}\sum_{i\in \{Case\},j\in \{Control\}}D_{ij}^{G_{1},G_{2}}}{\frac{1}{\left(\begin{array}{c} n_{CS}\\ 2 \end{array}\right)}\sum_{\begin{array}{c} i,j\in \{Case\}\\ i<j \end{array}}D_{ij}^{G_{1},G_{2}}+\frac{1}{\left(\begin{array}{c} n_{CN}\\ 2 \end{array}\right)}\sum_{\begin{array}{c} i,j\in \{Control\}\\ i<j \end{array}}D_{ij}^{G_{1},G_{2}}}\\ \approx \frac{{{\hat{p}}^{\prime }}_{\left(G_{1},G_{2}\right)}\Pi_{D_{\left(G_{1},G_{2}\right)}}{\hat{q}}_{\left(G_{1},G_{2}\right)}}{{{\hat{p}}^{\prime }}_{\left(G_{1},G_{2}\right)}\Pi_{D_{\left(G_{1},G_{2}\right)}}{\hat{p}}_{\left(G_{1},G_{2}\right)}+{{\hat{q}}^{\prime }}_{\left(G_{1},G_{2}\right)}\Pi_{D_{\left(G_{1},G_{2}\right)}}{\hat{q}}_{\left(G_{1},G_{2}\right)}}, \end{array}$$

where *n_CS _*and *n_CN _*are the numbers of cases and controls, respectively; ${\hat{p}}_{\left(G_{1},G_{2}\right)}$ and ${\hat{q}}_{\left(G_{1},G_{2}\right)}$ are the vectors of joint haplotype/genotype frequencies of genes *G*_1 _and *G*_2_, for the case and control samples, respectively; $\Pi_{D_{\left(G_{1},G_{2}\right)}}$ is the dissimilarity matrix of the joint haplotypes/genotypes of *G*_1 _and *G*_2 _(see Appendix I). When ${\hat{p}}_{\left(G_{1},G_{2}\right)}={\hat{q}}_{\left(G_{1},G_{2}\right)}$ (cases and controls have a same haplotype/genotype distribution), the test statistic is 0.5. When ${\hat{p}}_{\left(G_{1},G_{2}\right)}\neq {\hat{q}}_{\left(G_{1},G_{2}\right)}$, the test statistic is larger than 0.5. This statistic tests whether the average dissimilarity *between *cases and controls (between-group dissimilarity) is significantly large, with the adjustment of the dissimilarity *within *the case group and that within the control group (within-group dissimilarity). This is to mimic the *F *test to compare the between-group variability with the within-group variability. However, because of the complex correlation introduced by pair-wise similarities, the distribution of the test statistic is difficult to derive analytically, and permutation is required to obtain *P *values.

### Simulation Study

Simulation studies were conducted to evaluate the performance of our method. We extended the simulation scheme of Li et al. [18] to two chromosomal regions. In each region, 4,000 haplotypes across 300 kb were generated using the coalescent-based program ms [19]. The effective population size was set at 10,000, the recombination rate per base pair (bp) per generation was set at 10^-9^, and 300 SNPs were simulated in each region. For the human genome, recombination occurs at an average rate of about 10^-8 ^per bp per generation [20]. Our recombination rate, 10^-9 ^per bp per generation, is the low end of the recombination rates in the human genome [18], representing a stronger linkage disequilibrium (LD). We chose this rate because multi-marker approaches are primarily designed for strong-LD regions. In each chromosomal region, 2,000 diplotypes were generated by randomly pairing the 4,000 haplotypes. Then the 2,000 diplotypes of the first region were randomly paired with the 2,000 diplotypes of the second region, to form 2,000 subjects. In this way, we generated 300 datasets.

We then considered nine disease models listed in Additional file 1. Additional file 1 lists the causal allele frequencies, the penetrance values of two-locus genotypes, and the marginal penetrance values of one-locus genotypes, for all disease models. Model 0 was used to evaluate Type-I error rates, while the other eight models were used to evaluate powers. Models 1-6 exhibit interactions in the absence of main effects when genotypes conform to Hardy-Weinberg equilibrium. We used these six disease models because they further challenged the ability of our method to discover the joint associations (or 'interactions' in this situation) of gene pairs. Models 7 and 8 exhibit both interactions and main effects. Model 7 is the *jointly dominant-dominant model*, which requires at least one copy of the disease allele from both loci to be affected [21,22]. Model 8 has the same penetrance table with Model 3, but has different causal allele frequencies. We deliberately let the causal allele frequency of one locus be smaller than that of another locus.

For each dataset, we first randomly selected two SNPs (each from among 300 SNPs in a region) with similar MAFs to those of the causal SNPs (the tolerable difference was set to be 0.02), pretending them as the two causal SNPs. We then used the *H-clust *method [23,24] to choose tag SNPs with a subset formed by 200 subjects randomly drawn from the pool of 2,000 subjects. Tag SNPs were chosen with quality (MAF > 0.1) and correlation (the cut-off value for finding clusters was set to be 0.85). In each repetition, cases and controls were sampled with replacement from the pool of 2,000 subjects, where case/control status was assigned according to the genotypes of the two causal SNPs. After generating the phenotypes, the genotypes of the causal SNPs were removed from our datasets. Each chromosomal region was formed by eight SNPs - four to the left and four to the right of every causal SNP.

We evaluated the performance of our method with the matching measure ('MATCH') and the counting measure ('COUNT') of haplotypes. We also used two genotype similarity measures: 'IBS' and 'W-IBS'. We compared these with the *HapForest *approach [13]. *HapForest *is based on a tree structure, and is naturally suitable for analyzing interactions. Following the instructions of *HapForest*, we first invoked *SNPHAP *[25] to estimate the haplotype frequencies for each individual. Then *HapForest *was used to identify haplotypes and haplotype-haplotype interactions in association with the disease. This method suggests potential epistasis among significant haplotypes. For *HapForest*, a rejection of null hypothesis was defined as the identification of at least one significant haplotype from any of the two chromosomal regions.

The Pearson's *χ*^2 ^test was also performed for comparison, in which the joint haplotype distributions of the two chromosomal regions were compared between cases and controls. Rather than using the asymptotic *χ*^2 ^distribution, we randomly assigned the disease status in each permutation and determined the *P *value of observed *χ*^2 ^statistics. To calculate haplotype similarities from unphased multi-marker genotypes, we first inferred haplotype phases by the EM algorithm, using the function of 'haplo.em' in the 'haplo.stats' package [17]. The obtained posteriors were then treated as weights, and all possible haplotype pairs were considered with their probabilities (see equation (2)). All the haplotypes with frequencies less than 0.01 are considered to be rare haplotypes. To avoid possible genotyping errors, we follow Sha et al. [26] to merge each rare haplotype with its most similar common haplotype (see the modified EM algorithm proposed by Sha et al. [26]). For example, Haplotype A (1-1-1-2-1-1-1-1) is considered to be a rare haplotype because its frequency is less than 0.01. Haplotypes C (1-1-1-1-1-1-1-1) and F (1-1-1-2-2-1-1-1) are the most similar haplotypes to Haplotype A (both with a similarity of 0.875 by using the counting measure), and their haplotype frequencies are 0.2 and 0.1, respectively. We merge Haplotype A with Haplotype C, the most similar haplotype with the highest frequency. We then update the haplotype data by replacing Haplotype A with Haplotype C.

We also compared our methods with the tests for SNP × SNP epistasis by using case-control data or case-only data (with the --fast-epistasis command implemented by PLINK-1.07) [27]), hereafter referred to as 'CS-CN' and 'CS', respectively. In our simulation, each chromosomal region was formed by eight SNPs, and there were 64 tests for SNP × SNP epistasis. We recorded the minimum *P *value (*P*_min_) from among all the 64 *P *values, and then adjusted this *P*_min _on the basis of Sidak correction [28], with an effective number of tests, *M_eff_*. That is, we adjusted the minimum *P *value (*P*_min_) by $P_{\min,corrected,adjusted}=1-{\left(1-P_{\min}\right)}^{M_{eff}}$.

We then evaluated the validity and power of the eight tests with the 300 datasets. For each dataset, we recorded the *P *values of 50 repetitions (so there were 15,000 *P *values in total); in each repetition, *P *values were obtained with 1,000 permutations. Given a significance level, the type I error rate (if under Model 0) or power (if under Models 1-8) was the proportion of the number of *P *values smaller than the significance level to the total number of *P *values.

For CS-CN and CS, the *P *value used was $P_{\min,corrected,adjusted}=1-{\left(1-P_{\min}\right)}^{M_{eff}}$. The effective number of tests (*M_eff_*) was estimated by the eigenvalue-based approach [29,30]. For each subject, we had 8+8 genotype coding values (0, 1, or 2), and 64 pair-wise products of genotype coding values, one from a SNP in region 1 and another from a SNP in region 2. Based on *n *subjects, we obtained a 64 × 64 correlation matrix for these 64 pair-wise products of genotype coding values. Then the eigenvalues of this correlation matrix were calculated to estimate the effective number of tests (see [29,30], or see [31] for a nice review).

## Results

### Type-I Error Rates

**Figure 1:**
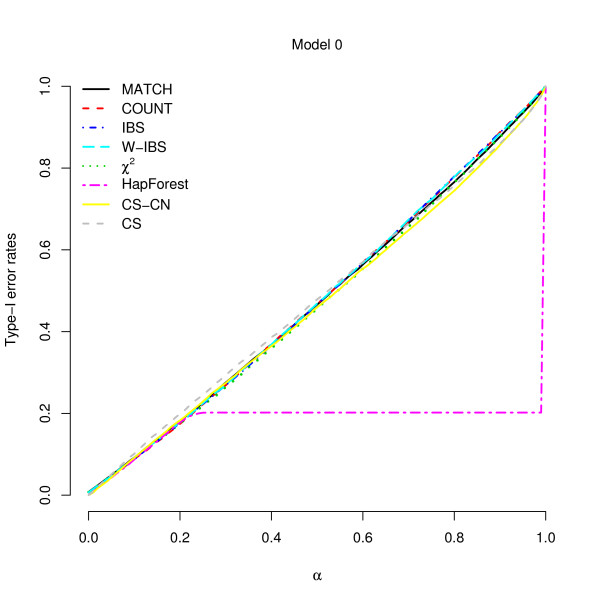
**Type-I error rates under different nominal significance levels**. The *x*-axis is nominal significance level, and the *y*-axis is type-I error rate.

In Additional file 1, Model 0 (disease status independent of the composite genotypes) was used to evaluate the type-I error rates. This model demonstrates our null hypothesis: no main effects and no interactions. In this model, the penetrance of each composite genotype was set to be 0.05. The sample size was set at 200 subjects, of which half were cases and half were controls. Figure 1 presents the type-I error rates under different nominal significance levels (*α*). For *α *smaller than 0.2, the type-I error rates of all the tests corresponded to the nominal significance levels (*α*), suggesting the validity of these tests. (For *α *larger than 0.2, the type-I error rates of *HapForest *failed to match with the nominal significance levels. *HapForest *reported *P *values as 1.0 when the association signal was not strong. However, this makes no influence on our following discussions because *α *is usually set at a small value.)

### Statistical Power

For all models except for Models 2 and 7, the total sample size was set at 1,000 subjects, of which half were cases and half were controls. For Models 2 and 7, the total sample size was set at 150 and 50, respectively. If the sample size was also set at 1,000 for Models 2 and 7, the powers of these tests would be all close to 1. Therefore, we chose two smaller sample sizes for effectively exploring the power difference between these tests. The power performances of these tests vary with the property of disease mutants introduced at rare/common haplotypes.

We first define two scores to distinguish the two situations. Let $SC_{1}=\sum_{j}I(f_{h_{j}}^{G_{1}}\geq 0.1)\times \sum_{k}I(f_{h_{k}}^{G_{2}}\geq 0.1)$ and $SC_{2}=\sum_{j}I(f_{h_{j}}^{G_{1}}\geq 0.2)\times \sum_{k}I(f_{h_{k}}^{G_{2}}\geq 0.2)$, where ***I***(·) is the indicator function, $f_{h_{i}}^{G_{g}}$ is the frequency of the *i*^th ^high-risk haplotype at *G_g_*, *g *= 1, 2. We estimated haplotype frequencies based on all 2,000 subjects in a dataset when calculating the scores of *SC*_1 _or *SC*_2_. While the score of *SC*_2 _is designed for Models 1-4 and 7, *SC*_1 _is designed for Models 5, 6, and 8 (because of their relatively low causal allele frequencies). Disease mutants were considered to be introduced at rare/common haplotypes if *SC*_2 _≤ 1/*SC*_2 _>1 (for Models 1, 2, 7); *SC*_2 _= 0/*SC*_2 _= 1 (for Models 3, 4); *SC*_1 _= 0/*SC*_1 _= 1 (for Models 5, 6); *SC*_1 _≤ 1/*SC*_1 _>1 (for Model 8).

**Figure 2:**
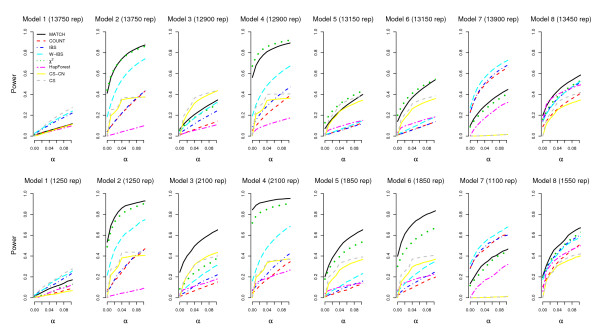
**Powers of the eight tests, stratified by the property of disease mutants introduced at rare/common haplotypes**. The *x*-axis is significance level, and the *y*-axis is power. The top row is for disease mutants introduced at rare haplotypes; the bottom row, at common haplotypes. The numbers shown in the parentheses are the numbers of repetitions summed from all the datasets with disease mutants introduced at rare/common haplotypes.

Figure 2 presents the powers of the eight tests when *α *is set to be smaller than 0.1, stratified by the property of disease mutants introduced at rare/common haplotypes. For most models, the two most powerful tests are our similarity method with the matching measure (MATCH) and the Pearson's *χ*^2 ^test. MATCH is more powerful than the Pearson's *χ*^2 ^test when the disease mutants were introduced at common haplotypes. Conversely, MATCH is less powerful than the Pearson's *χ*^2 ^test when the disease mutants were introduced at rare haplotypes. For Model 1, haplotype-perspective methods provide no power, while diplotype-perspective methods (IBS and W-IBS) and the test for SNP × SNP epistasis by using case-only data (CS) have better performances.

*HapForest *is not as powerful as MATCH and the Pearson's *χ*^2 ^test. *HapForest *suggests potential epistasis among significant haplotypes. At each step, it builds a classifier that optimally distinguishes cases from controls based on haplotype data. This divides the whole sample into smaller and smaller subgroups by maximizing the local optimality at each node. However, the combination of local optimalities does not assure us of an overall optimality [32].

The tests for SNP × SNP epistasis by using case-control data or case-only data (CS-CN and CS) are not powerful under most disease models. Although our disease status was influenced by the joint effects of two SNPs (see Additional file 1), the tests for SNP × SNP epistasis suffered from power loss because of the need of corrections for multiple testing.

COUNT and IBS often have similar performances, because similarity measure based on the number of alleles in common between haplotypes (COUNT) is similar to that based on the number of alleles in common between individuals (IBS). Model 1 is an exception, because haplotype-perspective methods would not present any power under this model (see the penetrance values of two-locus genotypes for Model 1). W-IBS is a counting measure inversely weighted by genotype frequencies, and it is more powerful than COUNT and IBS. For most models (Models 2-6 and 8), COUNT, IBS, and W-IBS are inferior to MATCH, because the counting measures are more sensitive to marker informativity and LD patterns (results not shown). For Model 7, it requires at least one copy of the disease allele from both loci to be affected. Because the disease status is influenced by the counts of disease alleles, methods with the counting measures (COUNT, IBS, and W-IBS) are more powerful.

## Discussion

Detecting joint associations of candidate genes responsible for common human diseases is a well-recognized issue. A candidate gene can contain many SNPs, and high-dimensionality becomes an important issue. The Pearson's *χ*^2 ^test and the tests for SNP × SNP epistasis suffer from power loss because of large numbers of degrees of freedom and the need of adjustment for multiple testing, respectively. Compared with these conventional association methods, similarity methods are less vulnerable to the penalty of high-dimensionality.

Some similarity methods have been proposed based on this consideration. Tzeng et al. [15] compared the case-case similarity with the control-control similarity, because haplotypes around a causal locus might be more similar in two cases than in two controls randomly selected from the population. However, as pointed out by Sha et al. [26], this consideration might not be very plausible for complex diseases which were presumed to be affected by many genes and gene-environment interactions. The similarity within controls is not necessarily smaller than that within cases, because controls could be more likely to share protective haplotypes. Therefore, Sha et al. [26] proposed a test statistic that compared the between-group similarity with the within-group similarity. Our test statistics is also based on this consideration. Our test and Sha et al.'s test [26] will have similar performances, given a same similarity measure.

In this paper, we use the product of similarities of two genes/regions as a new similarity measure, which can account for the joint association of the two genes/regions, including main effects and/or interactions. This new measure can be built in the similarity test statistic. Furthermore, our equation (3) can be used to test the main effects (see Appendix II) or the joint associations of gene triplets by using: $D_{ij}^{G_{1},G_{2},G_{3}}=1-S_{ij}^{G_{1},G_{2},G_{3}}=1-S_{ij}^{G_{1}}\times S_{ij}^{G_{2}}\times S_{ij}^{G_{3}}$.

The computational burden of our method is reasonable for real data analyses, although permutation is required to obtain *P *values. If there are 100 candidate genes (each with eight tag SNPs), there will be a total of 4,950 combinations of gene pairs. With our experiences in simulations, it might take two to three days to test the 4,950 combinations for approximately 1000 subjects, given an Intel Xeon workstation with four 2.0 GHz CPUs and 2.0 GB of memory.

In general, our similarity test with the matching measure (MATCH) and the Pearson's *χ*^2 ^test have better power performances. However, because both are haplotype-perspective methods, they are not appropriate for Model 1. Under this model, only the four heterozygous genotypes (*AA*-*Bb*, *Aa*-*BB*, *Aa*-*bb*, *aa*-*Bb*) lead to the disease. The implication is that besides the within-locus interference, there is some between-locus interference, and the two interferences cancel out [21] (so the double-heterozygosity genotype does not lead to the disease). The four heterozygous genotypes (*AA*-*Bb*, *Aa*-*BB*, *Aa*-*bb*, *aa*-*Bb*) generate four combinations of haplotypes: *AB *(one with allele *A *and one with allele *B*), *Ab*, *aB*, *ab*, with a same probability. Therefore, the four combinations of haplotypes are equally distributed in cases and in controls, and the haplotype-perspective methods cannot provide any power to this model.

The concept of testing joint associations can be used in the genomic distance-based regression [16]. Let ***D ***be the distance/dissimilarity matrix with elements: $D_{ij}^{G_{1},G_{2}}=1-S_{ij}^{G_{1},G_{2}}=1-S_{ij}^{G_{1}}\times S_{ij}^{G_{2}}$, and let ***X*** be the matrix containing information of phenotypes, which can be binary or continuous. Then the pseudo-*F *statistic can be used to test the association of phenotypic similarity with genetic similarity. The genomic distance-based regression [16] has the potential to adjust for covariate effects. With the need of adjusting for covariates, one can consider this approach with the joint similarities among genes.

## Conclusions

In detecting joint associations between disease and gene pairs, our similarity test is a complementary method to the Pearson's *χ*^2 ^test.

## Competing interests

The authors declare that they have no competing interests.

## Authors' contributions

W-Y L conceptualized the study, performed the simulation studies, and drafted the manuscript. W-C L provided advice and revised the manuscript. All authors read and approved the final manuscript.

## Appendix

### Appendix I: Derivation of equation (3)

$$\begin{array}{l} \frac{1}{\left(\begin{array}{c} n_{CS}\\ 2 \end{array}\right)}\sum_{\begin{array}{c} i,j\in \{Case\}\\ i<j \end{array}}D_{ij}^{G_{1},G_{2}}=\frac{2}{n_{CS}\times (n_{CS}-1)}\sum_{\begin{array}{c} i,j\in \{Case\}\\ i<j \end{array}}D_{ij}^{G_{1},G_{2}}=\frac{1}{n_{CS}\times (n_{CS}-1)}\sum_{i,j\in \{Case\}}D_{ij}^{G_{1},G_{2}}\\ =\frac{1}{n_{CS}^{2}}\sum_{i,j\in \{Case\}}D_{ij}^{G_{1},G_{2}}+\frac{1}{n_{CS}^{2}\times (n_{CS}-1)}\sum_{i,j\in \{Case\}}D_{ij}^{G_{1},G_{2}}\\ =\sum_{k,l,m,n}{\hat{p}}_{\left(G_{1_{k}},G_{2_{l}}\right)}\cdot {\hat{p}}_{\left(G_{1_{m}},G_{2_{n}}\right)}\cdot D(G_{1_{k}},G_{2_{l}};G_{1_{m}},G_{2_{n}})+\frac{1}{\left(n_{CS}-1\right)}\cdot \sum_{k,l,m,n}{\hat{p}}_{\left(G_{1_{k}},G_{2_{l}}\right)}\cdot {\hat{p}}_{\left(G_{1_{m}},G_{2_{n}}\right)}\cdot D(G_{1_{k}},G_{2_{l}};G_{1_{m}},G_{2_{n}})\\ ={{\hat{p}}^{\prime }}_{\left(G_{1},G_{2}\right)}\Pi_{D_{\left(G_{1},G_{2}\right)}}{\hat{p}}_{\left(G_{1},G_{2}\right)}+\frac{1}{\left(n_{CS}-1\right)}\cdot \left({{\hat{p}}^{\prime }}_{\left(G_{1},G_{2}\right)}\Pi_{D_{\left(G_{1},G_{2}\right)}}{\hat{p}}_{\left(G_{1},G_{2}\right)}\right)\\ ={{\hat{p}}^{\prime }}_{\left(G_{1},G_{2}\right)}\Pi_{D_{\left(G_{1},G_{2}\right)}}{\hat{p}}_{\left(G_{1},G_{2}\right)}+O\left(\frac{1}{n_{CS}}\right)\\ \approx {{\hat{p}}^{\prime }}_{\left(G_{1},G_{2}\right)}\Pi_{D_{\left(G_{1},G_{2}\right)}}{\hat{p}}_{\left(G_{1},G_{2}\right)}. \end{array}$$

Similarly, $\frac{1}{\left(\begin{array}{c} n_{CN}\\ 2 \end{array}\right)}\sum_{\begin{array}{c} i,j\in \{Control\}\\ i<j \end{array}}D_{ij}^{G_{1},G_{2}}\approx {{\hat{q}}^{\prime }}_{\left(G_{1},G_{2}\right)}\Pi_{D_{\left(G_{1},G_{2}\right)}}{\hat{q}}_{\left(G_{1},G_{2}\right)}$. We also have

$$\frac{1}{n_{CS}\times n_{CN}}\sum_{i\in \{Case\},j\in \{Control\}}D_{ij}^{G_{1},G_{2}}=\sum_{k,l,m,n}{\hat{p}}_{\left(G_{1_{k}},G_{2_{l}}\right)}\cdot {\hat{q}}_{\left(G_{1_{m}},G_{2_{n}}\right)}\cdot D(G_{1_{k}},G_{2_{l}};G_{1_{m}},G_{2_{n}})={\hat{p^{\prime }}}_{\left(G_{1},G_{2}\right)}\Pi_{D_{\left(G_{1},G_{2}\right)}}{\hat{q}}_{\left(G_{1},G_{2}\right)}.$$

Note that ${\hat{p}}_{\left(G_{1},G_{2}\right)}$ and ${\hat{q}}_{\left(G_{1},G_{2}\right)}$ are the vectors of joint haplotype/genotype frequencies of genes *G*_1 _and *G*_2_, for the case and control samples, respectively; ${\hat{p}}_{\left(G_{1_{k}},G_{2_{l}}\right)}$ is the joint frequency of the *k*th category of haplotype/genotype at *G*_1 _and the *l*th category of haplotype/genotype at *G*_2_; $\Pi_{D_{\left(G_{1},G_{2}\right)}}$ is the dissimilarity matrix of the joint haplotypes/genotypes at *G*_1 _and *G*_2_, where its element $D(G_{1_{k}},G_{2_{l}};G_{1_{m}},G_{2_{n}})$ is the dissimilarity between $\left(G_{1_{k}},G_{2_{l}}\right)$ and $\left(G_{1_{m}},G_{2_{n}}\right)$.

### Appendix II: Test for main effects

When testing for main effects, we use only one gene/region in equation (3), i.e.,

$$T=\frac{\frac{1}{n_{CS}\times n_{CN}}\sum_{i\in \{Case\},j\in \{Control\}}D_{ij}^{G_{g}}}{\frac{1}{\left(\begin{array}{c} n_{CS}\\ 2 \end{array}\right)}\sum_{\begin{array}{c} i,j\in \{Case\}\\ i<j \end{array}}D_{ij}^{G_{g}}+\frac{1}{\left(\begin{array}{c} n_{CN}\\ 2 \end{array}\right)}\sum_{\begin{array}{c} i,j\in \{Control\}\\ i<j \end{array}}D_{ij}^{G_{g}}}\approx \frac{{{\hat{p}}^{\prime }}_{\left(G_{g}\right)}\Pi_{D_{\left(G_{g}\right)}}{\hat{q}}_{\left(G_{g}\right)}}{{{\hat{p}}^{\prime }}_{\left(G_{g}\right)}\Pi_{D_{\left(G_{g}\right)}}{\hat{p}}_{\left(G_{g}\right)}+{{\hat{q}}^{\prime }}_{\left(G_{g}\right)}\Pi_{D_{\left(G_{g}\right)}}{\hat{q}}_{\left(G_{g}\right)}},$$

where $D_{ij}^{G_{g}}=1-S_{ij}^{G_{g}}$ and $S_{ij}^{G_{g}}$ can be calculated from haplotypes or genotypes; ${\hat{p}}_{\left(G_{g}\right)}$ and ${\hat{q}}_{\left(G_{g}\right)}$ are the vectors of haplotype/genotype frequencies at gene *G_g_*, for the case and control samples, respectively; $\Pi_{D_{\left(G_{g}\right)}}$ is the dissimilarity matrix of the haplotypes/genotypes at *G_g_*.

## Supplementary Material

Additional file 1 **Table S1**. The penetrance tables and causal allele frequencies of nine disease models. Click here for file
