# Supplementary material for: Discovering joint associations between disease and gene pairs with a novel similarity test
Source: BMC Genet. 2010 Oct 4;11:86. doi: 10.1186/1471-2156-11-86 (PMC2959050; doi:10.1186/1471-2156-11-86)
Supplement: Additional file 1 — Table S1. The penetrance tables and causal allele frequencies of nine disease models. [file 1471-2156-11-86-S1.DOC]

# Additional file 1 Table S1  The penetrance tables and causal allele frequencies of nine disease models (Models 1~6 are from Figure 2 of Ritchie et al. [1])

|  | **Model 0** | | |  |  |  |  | | |  |
| --- | --- | --- | --- | --- | --- | --- | --- | --- | --- | --- |
|  | *BB* | *Bb* | *bb* | Margin* |  |  |  |  |  |  |
| *AA* | 0.05 | 0.05 | 0.05 | 0.050 |  |  |  |  |  |  |
| *Aa* | 0.05 | 0.05 | 0.05 | 0.050 |  |  |  |  |  |  |
| *aa* | 0.05 | 0.05 | 0.05 | 0.050 |  |  |  |  |  |  |
| Margin | 0.050 | 0.050 | 0.050 |  |  |  |  |  |  |  |
|  | *P*(*A*)= *P*(*B*)=0.5 | | |  |  |  |  |  |  |  |
|  |  |  |  |  |  |  |  |  |  |  |
|  | **Model 1** | | |  |  |  | **Model 2** | | |  |
|  | *BB* | *Bb* | *bb* | Margin |  |  | *BB* | *Bb* | *bb* | Margin |
| *AA* | 0 | 0.10 | 0 | 0.050 |  | *AA* | 0 | 0 | 0.10 | 0.025 |
| *Aa* | 0.10 | 0 | 0.10 | 0.050 |  | *Aa* | 0 | 0.05 | 0 | 0.025 |
| *aa* | 0 | 0.10 | 0 | 0.050 |  | *aa* | 0.10 | 0 | 0 | 0.025 |
| Margin | 0.050 | 0.050 | 0.050 |  |  | Margin | 0.025 | 0.025 | 0.025 |  |
|  | *P*(*A*)= *P*(*B*)=0.5 | | |  |  |  | *P*(*A*)= *P*(*B*)=0.5 | | |  |
|  |  |  |  |  |  |  |  |  |  |  |
|  | **Model 3** | | |  |  |  | **Model 4** | | |  |
|  | *BB* | *Bb* | *bb* | Margin |  |  | *BB* | *Bb* | *bb* | Margin |
| *AA* | 0.08 | 0.07 | 0.05 | 0.059 |  | *AA* | 0 | 0.01 | 0.09 | 0.054 |
| *Aa* | 0.10 | 0 | 0.10 | 0.063 |  | *Aa* | 0.04 | 0.01 | 0.08 | 0.051 |
| *aa* | 0.03 | 0.10 | 0.04 | 0.062 |  | *aa* | 0.07 | 0.09 | 0.03 | 0.055 |
| Margin | 0.059 | 0.060 | 0.063 |  |  | Margin | 0.054 | 0.055 | 0.053 |  |
|  | *P*(*A*)= *P*(*B*)=0.25 | | |  |  |  | *P*(*A*)= *P*(*B*)=0.25 | | |  |
|  |  |  |  |  |  |  |  |  |  |  |
|  | **Model 5** | | |  |  |  | **Model 6** | | |  |
|  | *BB* | *Bb* | *bb* | Margin |  |  | *BB* | *Bb* | *bb* | Margin |
| *AA* | 0.07 | 0.05 | 0.02 | 0.026 |  | *AA* | 0.09 | 0.001 | 0.02 | 0.017 |
| *Aa* | 0.05 | 0.09 | 0.01 | 0.025 |  | *Aa* | 0.08 | 0.07 | 0.005 | 0.017 |
| *aa* | 0.02 | 0.01 | 0.03 | 0.026 |  | *aa* | 0.003 | 0.007 | 0.02 | 0.017 |
| Margin | 0.026 | 0.025 | 0.026 |  |  | Margin | 0.018 | 0.018 | 0.017 |  |
|  | *P*(*A*)= *P*(*B*)=0.1 | | |  |  |  | *P*(*A*)= *P*(*B*)=0.1 | | |  |
|  |  |  |  |  |  |  |  |  |  |  |
|  | **Model 7** | | |  |  |  | **Model 8** | | |  |
|  | *BB* | *Bb* | *bb* | Margin |  |  | *BB* | *Bb* | *bb* | Margin |
| *AA* | 0.10 | 0.10 | 0 | 0.075 |  | *AA* | 0.08 | 0.07 | 0.05 | 0.055 |
| *Aa* | 0.10 | 0.10 | 0 | 0.075 |  | *Aa* | 0.10 | 0 | 0.10 | 0.078 |
| *aa* | 0 | 0 | 0 | 0 |  | *aa* | 0.03 | 0.10 | 0.04 | 0.053 |
| Margin | 0.075 | 0.075 | 0 |  |  | Margin | 0.059 | 0.060 | 0.063 |  |
|  | *P*(*A*)= *P*(*B*)=0.5 | | |  |  |  | *P*(*A*)=0.25; *P*(*B*)=0.125 | | |  |

* The marginal penetrance values (the probability of disease given a one-locus genotype, without considering the genotype of the other locus) are calculated under Hardy-Weinberg equilibrium. Models 1~6 exhibit interactions in the absence of main effects when genotypes conform to Hardy-Weinberg equilibrium.

1. Ritchie MD, Hahn LW, Moore JH: **Power of multifactor dimensionality reduction for detecting gene-gene interactions in the presence of genotyping error, missing data, phenocopy, and genetic heterogeneity**. *Genet Epidemiol* 2003, **24**(2):150-157.
